# Freedom of the mind

**DOI:** 10.3389/fpsyg.2013.00538

**Published:** 2013-08-27

**Authors:** Zoran Josipovic

**Affiliations:** Contemplative Science Lab, Department of Psychology, New York UniversityNew York, NY, USA

**Keywords:** meditation, consciousness, nondual awareness, mindfulness, mind wandering

“Thinking or not thinking, both always perfect, neither ever stepping from the ground.”

Zen saying

Mind wandering, task-independent thought, spontaneous thinking, free associating, creative imagining are some of the terms used to describe what occurs to our mind when we notice its functioning, or when we allow it the freedom to be in its natural condition: open, aware and spontaneously fluid. That such mentation is being increasingly declared as undesirable, is perhaps a sign of the times: seduced by a promise of digital perfection and virtual reality, we are forgetting that life is a lived experience in which meaning unfolds through a process of being embodied, as our minds “wander” from the unconscious and preconscious, to fully conscious. Thus, the question is not whether we can be free of thoughts and perform with ever increasing efficiency, but whether we are authentic and present to all of our being, including the wandering mind. Those background spontaneous thoughts that at first introspection appear to be task-unrelated, may on a closer look reveal a deeper meaning, whose significance is both task-related and self-related, as authentic being contains both the extrinsic task-related and intrinsic self-related aspects of experience. To the extent that mind wandering serves a function of dissociating and avoiding the awareness of certain aspects of one's present experience, it could be said to be related to unhappiness. But making a simple causal link between mind-wandering and unhappiness ignores the larger situatedness of both as manifestations of self-meaning and self-organization.

The fact that our life is a participatory experience that contains a dimension of relatedness with environment and other human beings and which reveals an ongoing dynamic flux between self-referential and other-referential concerns, has been a challenge and a source of bewilderment for us, seemingly from the dawn of civilization. Our instinctive impulse is to manage this situation by creating rigidified constructs and boundaries that fragment experience into competing dualities of subject vs. object, self vs. other, in-group vs. out-group, good vs. bad. Yet such an instinctive solution tends to create a host of other problems and turns the existential unease into an outright suffering. Attempts at solution advocated over the centuries by the various contemplative traditions often include reductionist strategies for collapsing experience into one or the other pole of duality, either emphasizing the self-related (subjective), or other-related (objective) pole, while de-emphasizing and devaluing the other, sometimes even abolishing both in kind of non-conscious void (Sharma, 1987). These strategies have at times been seen as goals in themselves, other times as stages in the progress of meditation (Lutz et al., 2007). What they have in common is the approach of using various attentional and other cognitive control strategies to suppress the unwanted aspect of experience from arising. In doing so, these techniques inadvertently create yet another layer of fragmentation.

The dualistic structuring of experience may have an interesting parallel in the global organization of the cortex into the intrinsic default network involved in self-referential processing, and the extrinsic or task-positive network involved in perceiving and acting in the environment (Golland et al., 2007; Soddu et al., 2009). Increases in the activation and functional connectivity in the areas of default network have been found with meditations that emphasize the subjective pole of experience (Yamamoto et al., 2006; Travis et al., 2010; Lou et al., 2011), while increases in activations and functional connectivity in the areas of the task-related extrinsic network have been found with meditations that emphasize the objective pole of experience. Additionally, meditations that emphasize the objective pole of experience are often accompanied by a decrease of activation in the areas of default network, as well as increased functional segregation between the two networks (Pagnoni et al., 2008; Brewer et al., 2011; Kilpatrick et al., 2011).

The majority of meditation studies done in the past several years belong to this second category, including both focused attention (FA) and mindfulness meditations (OM). As a result, a prevalent view of the neuroscience of meditation is emerging, according to which the main effect of meditation is a suppression of the activity of the default network, and an increase in its functional segregation from the task-positive extrinsic network (Fell, 2012). This tallies with those interpretations of Buddhist philosophy that see the goal of meditation as abolishing of the sense of self or subjective reference (Austin, 2011). Since spontaneous thinking is primarily seen as being self-referential, such thinking is supposed to be abolished as well. On these views, much of cognitive and affective processing is believed to cease with successful meditation, and one's experience becomes reduced to present sensory awareness, which accommodates a self as an agent in the world, but not a self as a knower of its own states and contents (Christoff et al., 2011). In terms of the neuroscience, this view emphasizes the lateral extrinsic areas of the brain over the medial intrinsic ones, sometimes seeing the subcortical brainstem structures and thalamus as involved in representing a core phenomenal self. (Christoff et al., 2011; Vago and Silbersweig, 2012). It also relies on a somewhat reductionist view of the function of intrinsic default network, as a seat of the narrative autobiographical self (Farb et al., 2007). But as recent research shows, default network is also involved in much of what we regard as uniquely human consciousness, including self-awareness, future planning and making decisions about one's current personal state, constructing a scene from memory, as well as mind-wandering (Mason et al., 2007; Smallwood et al., 2008; Christoff et al., 2009; Andrews-Hanna et al., 2010; Preminger et al., 2011).

When the mind is allowed to be free, its spontaneous activity reveals two aspects: the arising and passing of thoughts and other mental contents, and the background nondual awareness—an open, awake cognizance that precedes conceptualization and intention, and contextualizes and unifies both extrinsic task-positive and intrinsic self-referential mental processes, without fragmenting the field of experience into opposing dualities. This background awareness appears in meditation to be self-same and unchanging, an empty cognizance devoid of content, while various sensory, affective, and cognitive contents, and the various states of arousal, appear to it as dynamic processes or, as a well-known metaphor states, like images in a mirror (Lama, 2004). Is this awareness merely a degree of integration of different aspects of experience? Taxonomies found in the nondual traditions, and the anecdotal accounts of meditation practitioners over many centuries, indicate that this might be a distinct level of consciousness, one that has not yet been conceptualized within the mainstream of neuroscience.

Mental states cultivated in meditation are quite complex and the neural signatures we have been able to detect so far are almost certainly only a small part of what is going on in the brain during such states. Meditation methods that attempt to suppress and control various aspects of experience, in addition to being unnecessarily arduous and potentially hazardous, may be violating basic principles of brain organization: the brain, and the cortex in particular, is not organized into discrete self-sufficient modules with singular functions that can be turned on and off as needed. Rather, it is a dynamic system where coalitions of neurons self-organize into globally distributed networks with intrinsic spontaneous fluctuations that optimize the processing of information in a context-dependent manner (Raichle, 2011; Baars et al., 2013). The relevance of this for understanding meditation research is that meditation techniques do not need to be limited to cognitive control strategies that fragment and suppress aspects of oneself. Rather, a meditation style that allows for the natural functioning of mind, and that recognizes the nondual ground, can reveal a more integrated and authentic being—an underlying natural unity of intrinsic and extrinsic aspects of experience in the context of nondual awareness.

Future research could further differentiate the neural mechanisms of nondual awareness from those of FA and open monitoring (Josipovic et al., 2012; Josipovic, in press) and explore the effects of nondual awareness on a variety of cognitive and affective processes, including mind wandering (Travis et al., 2002; Lutz et al., 2013). But our enthusiasm in this direction ought to be tempered by a warning from contemplative traditions that nondual awareness is not about improving oneself, but about realizing the innate freedom of authentic being (Longchenpa, 1975). Keeping in this spirit, future research could find ways to examine freedom and authenticity conferred by nondual awareness.
